# Green Tea and the Risk of Colorectal Cancer: Pooled Analysis of Two Prospective Studies in Japan

**DOI:** 10.2188/jea.15.118

**Published:** 2005-08-30

**Authors:** Yoshinori Suzuki, Yoshitaka Tsubono, Naoki Nakaya, Yayoi Koizumi, Yoko Suzuki, Daisuke Shibuya, Ichiro Tsuji

**Affiliations:** 1 Division of Epidemiology, Departments of Public Health and Forensic Medicine, Tohoku University Graduate School of Medicine.; 2 Division of Health Policy, School of Public Policy, Tohoku University.; 3 Miyagi Cancer Society.

**Keywords:** Tea, Colorectal Neoplasms, Prospective Studies, Japan

## Abstract

BACKGROUND: Although laboratory experiments suggest protective effects of green tea against colorectal cancer, few prospective cohort studies have been conducted.

METHODS: We conducted a pooled analysis of two prospective cohort studies among residents in Miyagi Prefecture in rural northern Japan. The first study started in 1984 and included 26,311 subjects. The second study started in 1990 and included 39,604 subjects. The subjects responded to a self-administered questionnaire including an item on green tea consumption. With 7 to 9 years of follow-up, 305 colon and 211 rectal cancers were identified in the two cohorts through record linkage to a regional cancer registry. We used Cox regression to estimate the hazard ratio (HR) of colorectal cancer according to the consumption of green tea with adjustment for potential confounders, and pooled the estimates obtained from each cohort by general variance-based method.

RESULTS: Multivariate pooled HRs for colon cancer associated with drinking 1-2, 3-4, and 5 or more cups of green tea per day, as compared with less than 1 cup per day, were 1.06 (95% confidence interval [CI] = 0.74-1.52), 1.10 (0.78-1.55), 0.97 (0.70-1.35), respectively (trend p = 0.81). Corresponding HRs for rectal cancer were 0.85 (95% CI = 0.56-1.29), 0.70 (0.45-1.08), 0.85 (0.58-1.23), respectively (trend p = 0.31).

CONCLUSIONS: Consumption of green tea was not associated with lower risk of colorectal cancer.

A potential anti-carcinogenic effect of green tea has drawn much interest in experimental and epidemiologic studies.^1^ A fairly large number of epidemiologic studies have investigated the protective association between green tea and gastric cancer,^2^^-^^6^ but a few studies have shown an inhibitory effect of green tea or tea catechin in the development of chemically-induced carcinoma of the colorectum as well as of the stomach. Sepcifically, in vitro experiments suggest that green tea polyphenols inhibit the formation of hetero­cyclic amines, which is formed during the cooking of meats and fish and is implicated in the development of colorectal cancer.^7^ Animal experiments also suggest that green tea polyphenols suppress colon-carcinogenesis induced by heterocyclic amines.^8^

Epidemiologically, five case-control studies and one prospective cohort study have examined the association between consumption of green tea and the risk of colorectal cancer.^5^^,^^9^^-^^13^ For colon cancer, a case-control study in Japan found a significant inverse association,^10^ while the other five studies found no significant relation.^5^^,^^9^^,^^11^^-^^13^ For rectal cancer, a case-control study in China found a significant inverse association,^12^ while the other five studies found no significant relation.^5^^,^^9^^-^^11^^,^^13^ The only prospective cohort study conducted among atomic bomb survivors of Hiroshima and Nagasaki, Japan, found no association between consumption of green tea and risk of colon or rectal cancer.^13^

To further examine the association between consumption of green tea and the risk of colorectal cancer, we conducted a pooled analysis of two population-based prospective cohort studies in Miyagi Prefecture in rural northern Japan.

## METHODS

### Study Cohort

Study designs of the two cohort studies have been described in detail elsewhere.^14^^,^^15^ Briefly, Cohort 1 started in January 1984, when we delivered a self-administered questionnaire to 33,453 men and women (40 years of age or older) in three municipalities of Miyagi Prefecture. Usable questionnaire were returned from 31,345 subjects (93.7%).^14^ For Cohort 2, we delivered a self-administered questionnaire from June through August 1990 to 51,921 men and women (40-64 years of age) in 14 municipalities of the Prefecture. Usable questionnaires were returned from 47,605 subjects (91.7%).^15^ Protocols for the two cohort studies were approved by the institutional review board of Tohoku University Graduate School of Medicine. We considered the return of the self-administered questionnaires signed by the subjects to imply their consent to participate in the studies.

### Exposure Data

The questionnaires asked about “recent” (Cohort 1) or “usual” (Cohort 2) consumption of green tea. The two questionnaires used the same five categories: never, occasionally, 1-2 cups per day, 3-4 cups per day, 5 or more cups per day. “Never” and “occasionally” categories were collapsed into the single category “less than one cup per day” for the purpose of this analysis.

To examine the reproducibility and validity of the questionnaire measurements of green tea intake, we collected 12-day diet records from 56 men and 60 women who lived in the two municipalities in the study district.^16^ Spearman correlation coefficients between green tea consumption assessed by the Cohort 2 questionnaire and that measured by the diet records were 0.71 in men and 0.53 in women. Spearman correlation coefficients between green tea intake measured by the two questionnaires administered 12 month apart were 0.63 in men and 0.64 in women.

### Follow-up

For both Cohorts, we used population registries of the municipalities to obtain information on the vital and residential status of the subjects. We ascertained the incidence of cancer using the Miyagi Prefectural Cancer Registry covering the study areas.^17^ In this cancer registry, the proportion of colon cancer cases for which information was available only from death certificates was 3% in men and 7% in women, the proportion of rectal cancer cases for which information was available only from death certificates was 2% in men and 4% in women.^17^ Follow-up was conducted from January 1, 1984, through December 31, 1992, for Cohort 1, and from August 1, 1990, through March 31, 1997, for Cohort 2. The proportions of subjects who were lost to follow-up were 18.5% for Cohort 1, and 3.9% for Cohort 2. We defined subjects who were lost to follow-up as those who moved out of the study areas during follow-up period, and we assumed that subjects who had not have died or lost to follow-up remain alive in the study areas. In analysis, lost to follow-up was treated as censoring case.

We excluded cancer cases prevalent at baseline (541 in Cohort 1, and 1,110 in Cohort 2). We also excluded the subjects who did not respond to the question on green tea (4,493 in Cohort 1, and 6,891 in Cohort 2). Consequently, our analysis included 26,311 subjects with 269 colorectal cancer cases (158 colon and 111 rectum) in Cohort 1, and 39,604 subjects with 247 with colorectal cancer cases (147 colon and 100 rectum) in Cohort 2.

### Statistical Analysis

We counted person-year of follow-up for each subject from the beginning of follow-up until the date of diagnosis of colorectal cancer, the date of death, or the end of the follow-up period, whichever occurred first. Total person-years accrued were 200,039 for Cohort 1 and 290,836 for Cohort 2.

We estimated hazard ratios (HRs) and their 95% confidence intervals (CIs) of colon and rectal cancer according to the level of green tea consumption. We used Cox proportional-hazards regression to adjust for potentially confounding variables. For the two Cohorts, we considered the following variables as potential confounders: sex, age, family history of colorectal cancer, smoking status, body mass index (kg/m^2^), alcohol consumption, and consumption frequencies of black tea and coffee. For Cohort 1, we further adjusted for consumption frequencies of meat, green or yellow vegetables, other vegetables and fruits. For Cohort 2, we further adjusted for consumption frequencies of beef, pork, ham, liver, spinach, carrot and pumpkin, tomato, orange, other fruits, and juice.

To obtain a summary measure of results from Cohort 1 and Cohort 2, the general variance-based method was used to combine each HR and 95% CI.^18^ P values for the test of linear trend were calculated by treating the green tea consumption category as an ordinal variable. All reported P values are two-tailed.

## RESULTS

Table 1 compares the characteristics between subjects who consumed less than one cup per day and those who consumed five or more cups of green tea per day. For both Cohorts, men who consumed five or more cups per day were older, more likely to be current smokers and to consume black tea, less likely to consume coffee, as compared with the men who consumed less than one cup per day.

**Table 1.** Characteristics of the subjects according to green tea consumption.

	Men				Women				
	
	Cohort 1		Cohort 2		Cohort 1			Cohort 2	
			
	Green tea consumption (cups / day)				Green tea consumption (cups / day)				
	
	<1	5+	<1	5+	5+	5+	5+		5+
Characteristics									

No.	2,253	4,870	5,640	4,782	4,782	4,782	4,782		4,782

Age (yr), mean ± standard deviation	56.3 ± 11.3	57.6 ± 10.8	50.5 ± 7.5	53.7 ± 7.4	53.7 ± 7.4	53.7 ± 7.4	53.7 ± 7.4		53.7 ± 7.4

Smoking (%)									
Never	27.9	18.8	21.1	17.1	17.1	17.1	17.1		17.1
Past	23.8	23.2	20.2	20.8	20.8	20.8	20.8		20.8
Current (1-19 cigarettes / day)	18.9	17.2	16.2	13.5	13.5	13.5	13.5		13.5
Current (20+cigarettes / day)	29.4	40.9	42.6	48.7	48.7	48.7	48.7		48.7

Alcohol drinkig (%)									
Never	16.3	15.6	15.9	17.7	17.7	17.7	17.7		17.7
Past	10.6	8.0	7.2	8.0	8.0	8.0	8.0		8.0
Current	73.1	76.4	76.9	74.4	74.4	74.4	74.4		74.4

Body mass index (%)									
<18.5	5.2	4.4	1.9	2.2	2.2	2.2	2.2		2.2
18.5-24.9	72.3	73.4	70.5	68.5	68.5	68.5	68.5		68.5
25.0+	22.5	22.2	27.7	29.3	29.3	29.3	29.3		29.3

Daily beverage consumption (%)									

Black tea (cups / day)									
Never	65.0	47.6	64.9	59.8	59.8	59.8	59.8		59.8
Occasionally	31.3	44.8	33.2	36.8	36.8	36.8	36.8		36.8
1-2	2.9	5.6	1.5	2.3	2.3	2.3	2.3		2.3
3+	0.8	1.9	0.3	1.1	1.1	1.1	1.1		1.1

Coffee (cups / day)									
Never	34.7	18.0	20.6	19.8	19.8	19.8	19.8		19.8
Occasionally	35.2	46.5	32.9	41.7	41.7	41.7	41.7		41.7
1-2	19.5	25.2	29.0	24.6	24.6	24.6	24.6		24.6
3+	10.6	10.4	17.5	13.9	13.9	13.9	13.9		13.9

In Cohort 1, women who consumed five or more cups per day were older, more likely to be current smokers, drinkers and to consume black tea, less likely to consume coffee, as compared with the women who consumed less than one cup per day. In Cohort 2, women who consumed five or more cups per day were older, more likely to be current smokers and to consume black tea, less likely to be current alcohol drinkers and to consume coffee, as compared with the women who consumed less than one cup per day.

Table 2 presents the HRs for colon and rectal cancer according to green tea consumption. After adjustment for sex and age, green tea consumption was not associated with the risk of colon cancer in Cohort 1, Cohort 2, and the two Cohorts combined. Multivariate adjustment or the exclusion of cases of colon cancer diagnosed in the first three years of follow-up did not change the findings materially.

**Table 2.** Hazard ratios (HRs) and 95% confidence intervals (CIs) of colon and rectum cancer accoding to green-tea consumption.*

	Green-tea consumption (cups/day)				P for trend

	<1	1 or 2	3 or 4	5+	
Colon					
No.of cases / person-year of follow-up					
Cohort 1	26/36,642	30/34,157	36/43,780	66/85,460	
Cohort 2	39/84,398	29/68,774	36/62,753	43/74,911	

Sex- and age-adjusted HR					
Cohort 1	1.0	1.25 (0.74-2.11)	1.13 (0.68-1.86)	1.02 (0.65-1.61)	0.79
Cohort 2	1.0	0.93 (0.58-1.51)	1.11 (0.71-1.75)	0.94 (0.61-1.45)	0.93
Pooled	1.0	1.06 (0.75-1.52)	1.12 (0.80-1.56)	0.98 (0.71-1.34)	0.80

Multivariate HR1					
Cohort 1	1.0	1.19 (0.70-2.03)	1.08 (0.65-1.80)	1.03 (0.65-1.64)	0.87
Cohort 2	1.0	0.96 (0.59-1.57)	1.12 (0.70-1.77)	0.93 (0.59-1.46)	0.85
Pooled	1.0	1.06 (0.74-1.52)	1.10 (0.78-1.55)	0.97 (0.70-1.35)	0.81

Multivariate HR2					
Cohort 1	1.0	1.16 (0.65-2.08)	0.88 (0.49-1.58)	0.82 (0.49-1.39)	0.27
Cohort 2	1.0	1.01 (0.57-1.78)	1.23 (0.72-2.11)	0.84 (0.48-1.45)	0.67
Pooled	1.0	1.08 (0.72-1.62)	1.05 (0.71-1.57)	0.83 (0.57-1.21)	0.27

Rectum					
No.of cases / person-year of follow-up					
Cohort 1	18/36,642	20/34,157	22/43,780	51/85,460	
Cohort 2	38/84,398	20/68,774	15/62,753	27/74,911	

Sex- and age-adjusted HR					
Cohort 1	1.0	1.20 (0.63-2.27)	1.01 (0.54-1.88)	1.17 (0.68-2.00)	0.68
Cohort 2	1.0	0.65 (0.38-1.12)	0.48 (0.27-0.88)	0.64 (0.39-1.05)	0.05
Pooled	1.0	0.84 (0.56-1.29)	0.69 (0.45-1.06)	0.84 (0.58-1.21)	0.27

Multivariate HR1					
Cohort 1	1.0	1.34 (0.70-2.56)	1.14 (0.60-2.15)	1.34 (0.77-2.33)	0.40
Cohort 2	1.0	0.62 (0.36-1.06)	0.44 (0.24-0.82)	0.57 (0.34-0.95)	0.02
Pooled	1.0	0.85 (0.56-1.29)	0.70 (0.45-1.08)	0.85 (0.58-1.23)	0.31

Multivariate HR2					
Cohort 1	1.0	1.09 (0.49-2.41)	1.00 (0.46-2.15)	1.31 (0.68-2.51)	0.39
Cohort 2	1.0	0.54 (0.27-1.07)	0.39 (0.18-0.84)	0.66 (0.37-1.20)	0.14
Pooled	1.0	0.73 (0.43-1.22)	0.62 (0.36-1.07)	0.90 (0.58-1.40)	0.67

*: The multivariate hazard ratio (HR) has been adjusted for sex; age; family history of colorectal cancer; cigarette smoking; alcohol consumption; body mass index in kg / m^2^ (<18.5, 18.5-24.9, 25.0+ ); consumption of black tea, and coffee. The multivariate HR for cohortl has also been adjusted for consumption of meat, green-yellow vegetables, other vegetables, and fruits. The multivariate HR for Cohort2 has also been adjusted for consumption of beef, pork, ham, chicken, liver, spinach, carrot or pumpkin, tomato, orange, other fruits, and juice. HR1 denotes the relative risk with all cases of colorectal cancer included in the multivariate analysis, and HR2 the relative risk with cases diagnosed in the first three years of follow-up excluded from the analysis.

95% confidence intervals in parentheses.

After adjustment for sex and age, green tea consumption was not associated with risk of rectal cancer in Cohort 1, but was associated with lower risk of rectal cancer in Cohort 2. When the two Cohorts were pooled, however, green tea consumption was not associated with risk of rectal cancer. Null results in the pooled analysis did not change materially with multivariate adjustment or with the exclusion of cases of rectal cancer diagnosed in the first three years of follow-up.

We conducted stratified analyses according to sex, age, cigarette smoking, alcohol consumption, body mass index, and family history of colorectal cancer. Table 3 presents the pooled HRs stratified by sex and alcohol consumption. For colon cancer, we found no different results by sex and alcohol consumption. For rectal cancer, green tea consumption was associated with a decreased risk among men and current drinkers, but not among women and nondrinkers. We did not observe different findings for other variables.

**Table 3.** Pooled multivariate hazard ratios (HRs) and 95% confidence intervals (CIs) of colorectal cancer according to green tea consumption.

			Green-tea consumption (cups/day)				P for trend

			<1	1 or 2	3 or 4	5+	
			Colon				
Sex*	Men	No. of cases	36	39	46	64	
		Pooled multivariate HR	1.0	1.32 (0.83-2.10)	1.35 (0.86-2.12)	1.12 (0.72-1.74)	0.69

	Women	No. of cases	29	20	26	45	
		Pooled multivariate HR	1.0	0.78 (0.43-1.40)	0.78 (0.45-1.35)	0.79 (0.49-1.29)	0.34

Alcohol consumption**	Current drinkers	No. of cases	33	37	38	52	
		Pooled multivariate HR	1.0	1.30 (0.80-2.10)	1.16 (0.72-1.87)	0.98 (0.61-1.57)	0.77

	Nondrinkers	No. of cases	26	16	23	42	
		Pooled multivariate HR	1.0	0.73 (0.39-1.37)	0.82 (0.46-1.46)	0.86 (0.52-1.44)	0.71

			Rectum				
Sex*	Men	No. of cases	37	27	19	36	
		Pooled multivariate HR	1.0	0.85 (0.50-1.45)	0.58 (0.32-1.04)	0.62 (0.38-1.02)	0.02

	Women	No. of cases	19	13	18	42	
		Pooled multivariate HR	1.0	0.81 (0.40-1.66)	0.95 (0.48-1.89)	1.30 (0.70-2.42)	0.23

Alcohol consumption**	Current drinkers	No. of cases	32	24	16	34	
		Pooled multivariate HR	1.0	0.85 (0.49-1.49)	0.52 (0.28-0.97)	0.64 (0.38-1.09)	0.05

	Nondrinkers	No. of cases	15	13	14	30	
		Pooled multivariate HR	1.0	0.96 (0.42-2.22)	0.87 (0.36-2.10)	1.17 (0.56-2.42)	0.54

*: The pooled multivariate hazard ratio (HR) has been adjusted for age; family history of colorectal cancer; cigarette smoking; alcohol consumption; body mass index in kg / m^2^ (<18.5, 18.5-24.9, 25.0+ ); consumption of black tea, and coffee.

**: The pooled multivariate hazard ratio (HR) has been adjusted for sex; age; family history of colorectal cancer; cigarette smoking; body mass index in kg / m^2^ (<18.5, 18.5-24.9, 25.0+ ); consumption of black tea, and coffee.

The multivariate HR for cohort1 has also been adjusted for consumption of meat, green-yellow vegetables, other vegetables, and fruits.

The multivariate HR for cohort2 has also been adjusted for consumption of beef, pork, ham, chicken, liver, spinach, carrot or pumpkin, tomato, orange, other fruits, and juice.

95% confidence intervals in parentheses.

## DISCUSSION

In this pooled analysis of two population-based, prospective cohort studies in rural northern Japan, we generally found no association between green tea consumption and the risk of colorectal cancer. Green tea intake was associated with lower risk of rectal cancer among men and alcohol drinkers, but not among women and nondrinkers. Green tea was not related with lower risk of colon cancer irrespective of subjects’ sex or drinking status.

Five case-control studies and 1 prospective cohort study have examined the association between green tea and risk of colorectal cancer.^5^^,^^9^^-^^13^ For colon cancer, a case-control study in Japan found a significant inverse association,^10^ while other studies found no significant relation.^5^^,^^9^^-^^11^^,^^13^ For rectal cancer, a case-control study in China found a significant inverse association,^12^ while other studies found no significant relation.^5^^,^^9^^-^^11^^,^^13^ The inverse association observed in the two case-control studies may be partly due to recall bias.

Our study had several methodological advantages over prior studies of green tea and the risk of colorectal cancer, which include the use of prospective design, the use of validated food frequency questionnaire, and the large number of colorectal cancer cases accrued.

We observed significantly lower risk of rectal cancer associated with higher green tea intake among Cohort 2, men and alcohol drinkers, but not among Cohort 1, women or nondrinkers. Pooling of the results from the two Cohorts may require caution in interpretation, because a statistical test for the heterogeneity of the two Cohort results was not significant for sex- and age-adjusted HRs (p>0.05) but for multivariate HRs (p<0.05). Although it is possible that green tea is protective against rectal cancer only among these subgroups of subjects, chance may be a more likely explanation for the discrepancies according to the subgroups.

Although all of the six previous studies of green tea and colorectal cancer included both men and women,^5^^,^^9^^-^^13^ only one case-control study in China^12^ reported sex-specific results: green tea intake was significantly associated with lower risk of rectal cancer in men and women, while it was not significantly associated with risk of colon cancer in men or women. None of the previous studies reported the results of analyses stratified by categories of alcohol consumption. Future studies of green tea and colorectal cancer should report the findings stratified by sex and drinking status.

We used self-reports for a measure of green-tea consumption. In a validation study of the food frequency questionnaire in which 119 subjects provided four 3-day food records in one year and then responded to the questionnaire, we observed a reasonably high degree of validity and reproducibility for the questionnaire measurement of green tea intake; Spearman’s correlation coefficient for green-tea intake measured by the questionnaire and by the food records was 0.66, and the correlation between the two questionnaires administered in 6-month interval was 0.66.^14^

The proportion of subjects who were lost to follow-up was 18.5% for Cohort 1. The subjects who were lost to follow-up were more likely to be young, be current smokers, have family history of colorectal cancer, and less likely to be current alcohol drinkers, obese, as compared with those who could be follow-up.

As a potential limitation of the study, we could not specifically examine the effect of very high consumption of green tea, since the highest category in our questionnaire was five or more cups per day and we could not divide this category into subcategories (such as 5-9 cups and 10 or more cups). However, the validation study of our food frequency questionnaire found that 53% of the subjects who claimed to consume five or more cups per day in the questionnaire actually consumed seven or more cups per day according to 12-day diet records. This result suggests that a substantial proportion of our subjects in the highest consumption category actually consumed very high amounts of green tea. It is therefore unlikely that we failed to detect a large decrease in the risk of colorectal cancer associated with very high consumption of green tea.

We observed lower consumption of green tea in Cohort 2 than in Cohort 1 subjects. The most likely explanation would be the actual difference in green-tea intake between the two cohorts. Different validity of the questionnaire among the two cohorts may be another possibility, but it is unlikely that the different validity of the questionnaire lead to the different observations only for rectal cancer (inverse association in Cohort 2 and no association in Cohort 1) but not for colon cancer (no association in both Cohorts).

In conclusion, our pooled analysis of two population-based prospective cohort studies conducted in rural Japan showed no overall association between the consumption of green tea and the risk of colorectal cancer. The inverse association for rectal cancer observed in subgroup analyses (Cohort 1, men, and current drinkers) warrants further investigations.
